# Optimisation of Embryonic and Larval ECG Measurement in Zebrafish for Quantifying the Effect of QT Prolonging Drugs

**DOI:** 10.1371/journal.pone.0060552

**Published:** 2013-04-08

**Authors:** Sundeep Singh Dhillon, Éva Dóró, István Magyary, Stuart Egginton, Attila Sík, Ferenc Müller

**Affiliations:** 1 School of Clinical and Experimental Medicine, University of Birmingham, Birmingham, United Kingdom; 2 Department of Nature Protection, University of Kaposvar, Kaposvar, Hungary; 3 School of Biomedical Sciences, University of Leeds, Leeds, United Kingdom; National University of Singapore, Singapore

## Abstract

Effective chemical compound toxicity screening is of paramount importance for safe cardiac drug development. Using mammals in preliminary screening for detection of cardiac dysfunction by electrocardiography (ECG) is costly and requires a large number of animals. Alternatively, zebrafish embryos can be used as the ECG waveform is similar to mammals, a minimal amount of chemical is necessary for drug testing, while embryos are abundant, inexpensive and represent replacement in animal research with reduced bioethical concerns. We demonstrate here the utility of pre-feeding stage zebrafish larvae in detection of cardiac dysfunction by electrocardiography. We have optimised an ECG recording system by addressing key parameters such as the form of immobilization, recording temperature, electrode positioning and developmental age. Furthermore, analysis of 3 days post fertilization (dpf) zebrafish embryos treated with known QT prolonging drugs such as terfenadine, verapamil and haloperidol led to reproducible detection of QT prolongation as previously shown for adult zebrafish. In addition, calculation of Z-factor scores revealed that the assay was sensitive and specific enough to detect large drug-induced changes in QTc intervals. Thus, the ECG recording system is a useful drug-screening tool to detect alteration to cardiac cycle components and secondary effects such as heart block and arrhythmias in zebrafish larvae before free feeding stage, and thus provides a suitable replacement for mammalian experimentation.

## Introduction

Repolarisation abnormalities can lead to various conditions, such as cardiac arrhythmias and QT prolongation (where the duration of ventricular depolarisation and repolarisation is extended). Repolarisation in human ventricles occurs mainly *via* the voltage-gated potassium channel (hERG) in phase 3 of the ventricular myocyte action potential [1]. Therefore, disruption to this ion channel can result in QT prolongation, which is often a predisposing factor to arrhythmias and can be monitored through electrophysiological recordings. The cause of QT prolongation may be genetic (e.g. long QT syndrome) or drug-induced (e.g. anti-histamine terfenadine) and can lead to the development of tachycardia (Torsade de Pointes; [1]). Cardiac arrhythmias originate from abnormal electrical activity in the heart and may be life-threatening (leading to sudden cardiac arrest) or merely irritating (palpitations). Particularly, arrhythmias resulting from drug overdose can be fatal if left untreated, with sudden cardiac deaths accounting for approximately 450,000 deaths in the USA every year [2]. However, the mechanisms of many arrhythmias in the clinical setting remain poorly understood [3]. QT prolongation has received greater regulatory attention in recent years and there is a growing demand to find ways to counteract this problem using novel screens [4] [5]. Particularly, drug-induced QT prolongation is a growing problem and accounts for around 20% of failures in the drug development process, as it can be caused by many compounds that are often unrelated to each other in structure and function. Additionally, it can also occur by various mechanisms, such as blocking of cardiac ion channels such (e.g. hERG), either when they are inactive/active or open/closed [6]. Many compounds like terfenadine were only found to be QT prolonging post-marketing, as the old drug development standards were not able to detect confounding factors [7]. Therefore, pharmaceutical companies and regulatory agencies addressed this problem by developing *in silico* models and by introducing new standards in the drug development program [8].

Repolarisation of the myocardium is complex with several ion channels and various other components, such as cytoskeletal elements and receptors, playing an important role. This makes it difficult to identify the specific components that are affected by either drugs or genetic defects, which has raised the importance of zebrafish as a suitable model due to its electrophysiological similarity to humans [9]. The characteristics of cardiac myocyte action potentials from zebrafish closely resemble those of human myocytes, as orthologues of the cardiac ion channels found in humans exist in zebrafish, e.g. zERG (orthologue of human hERG) [10]. Although the zebrafish heart is two-chambered, it does have a coronary vasculature with the heart rate (120–180 bpm) and QT intervals (400–500 ms) similar to those observed in humans, whereas in mice the heart rate is much higher (300–600 bpm) and QT intervals are shorter (50 ms) [11] [12].

Zebrafish heart development occurs very rapidly, already beating 24 hours post-fertilisation, thus cardiac activity can be assessed relatively early compared to other animal models [13] [14]. Zebrafish produce a high number of rapidly developing embryos and therefore provide an ideal *in vivo* model for genetic, pharmacological and toxicological high throughput (HT) vertebrate screens [15] [16]. A number of reports describe HT screening of compounds demonstrating the potential of zebrafish in such assays [17] [18]. Using zebrafish embryos (referred to here as 0–5 dpf (days post fertilisation)) has several advantages for specifically detecting drug effects on the heart: *1)* small incubation volume is needed; *2)* animals can be kept alive for days without changing the solution, allowing relatively long-term studies; *3)* diffusion of chemicals can occur through the skin; *4)* embryos are not regulated under the UK Scientific Procedure Act 1986 and European Directive 2010/63/EU, and thus carry reduced bioethical limitations. Importantly, as zebrafish have similar electrical properties to the mammalian heart and appear to respond very similarly to drug treatment as humans [2] [16], they have been proposed as a useful model for drug toxicity testing [16] [19] [20]. Recently, cardiac repolarisation was used as a phenotypic readout in a drug-sensitised zebrafish screen for repolarisation genes [21], however this was restricted to manual measurement of action potential duration without an attempt at scaling up the electrophysiology technology.

Measuring the electrocardiogram of zebrafish embryos (5 dpf) using a single glass electrode has been demonstrated [20] and applied to recording ECGs in zebrafish larvae (7 dpf) following drug treatment with QRS prolonging agents [22]. An adult zebrafish ECG recording system has also been established and been shown to be useful to screen for QT prolongation, cardiac injury and heart regeneration [2] [19] [23]. An *in vitro* ECG method [24] and a flexible microelectrode array ECG system [25] have also been recently developed. However, all these methods have been developed for adult fish or late stage larvae that are subjected to animal experimentation licencing, and therefore incur regulatory restrictions in their use.

For embryos and pre free-feeding stage larvae, the high throughput methods developed thus far have been limited to detecting the heartbeat in zebrafish embryos using video recording [26] [27] and optical mapping [28]. One recent method developed was the visualisation of voltage dynamics in the zebrafish heart [29], which was able to detect cardiac dysfunction following application of the QT prolonging drug astemizole on 3 dpf embryos. However, as with video recording this method lacks the temporal and dynamic resolution required for analysis of components within the cardiac cycle that can be obtained by ECG, and thus is limited in data collection and utility of analysis. Taken together, while proof of principle ECG detection in embryonic and early stage larval zebrafish has been demonstrated, a robust method suitable for drug screening purposes is lacking. Therefore, we have optimised a method of ECG detection in early stage zebrafish larvae and demonstrated its utility in detecting the specific effect of QT prolonging and other cardiotoxic drugs.

## Materials and Methods

### Zebrafish Maintenance and Embryo Collection

Wild-type AB* zebrafish were maintained using standard conditions according to the UK Animals (Scientific Procedures) Act of 1986 in a flow-through system of aerated, charcoal filtered tap water with a 12 hour light/dark cycle (Tecniplast; UK). Breeding pairs were set up in breeding cages the day before collection of embryos. Embryos were obtained from crosses the following morning and then transferred immediately to E3 embryo medium (Sigma Aldrich), which was changed daily and maintained at 28°C.

### ECG Recording Procedure

For all experiments, except the anaesthetic and tubocurarine experiments, fish larvae were anaesthetised in ethyl-3 aminobenzoate methanesulfonate (0.3 mg/ml; Sigma) for 5–10 mins before transfer into 3 ml of embryo medium in the plate used for subsequent measurements. Once anaesthetised, one embryo was then transferred to the ECG recording plate (mini Petri dish with an unscented paraffin wax surface, containing 3 ml of fresh E3 embryo medium without MESAB). The embryo was positioned ventrally within a groove in the wax, and the tip (2 µm diameter) of a filled pre-pulled borosilicate glass micropipette (P84, World Precision Instruments) was positioned on the skin surface between the ventricle and atrium (no penetration) using micromanipulators (Narishige) and Inchworm step motors (Burleigh), viewed under a Nikon microscope (SMZ600). The micropipettes were filled with MicroFil (P85, World Precision Instruments) in 3 M potassium acetate solution (Sigma) and coloured with methylene blue (Sigma) to easier visualise the tips during recordings. A chloridised silver wire which carried the electrical signal to the amplifier was inserted into the micropipettes and a second reference electrode placed in the surrounding medium during recordings. The differential amplifier (NPI electronics) used for recording was operated in DC mode with the high pass filter set at 0.1 Hz. The raw ECG signals were digitised (PowerLab; ADI Instruments) and viewed using LabChart 7 (ADI Instruments).

Non-invasive recordings were taken by applying slight pressure over the surface of the heart using the tip of a glass capillary. Invasive recordings were taken by increasing the speed and also the distance moved by the step motor to puncture the skin over the heart. All of the recording equipment was housed on an air table within a grounded Faraday cage to minimise background noise. Experiments were performed at room temperature (21°C) except when otherwise stated. Temperature was controlled using a sensor placed in the recording plate and a homemade heating element (Cryocon 24 temperature controller). For optimisation experiments, recordings were taken for 5 mins. For the temperature experiments, embryos were initially acclimatised to each temperature for 30 mins before taking a recording of 5 mins. For drug treatments, ECG recordings were taken continuously after drug addition. A 5 mins segment before drug addition and a 15 minute segment where QT prolongation was first apparent by visual observation were selected for analysis. Recording lengths varied depending on concentration of drug used, e.g. for terfenadine treatment at 0.1, 0.3 and 1 µM recordings were taken for up to 1 hour or more, and for terfenadine at 50 µM recordings were taken for 40 minutes.

### Drug preparation

Cromakalim (a K_ATP_ channel opener [30]) and verapamil (an anti-arrhythmic [31]) were obtained from Tocris Bioscience. Haloperidol (a butyrophenone anti-psychotic [32]), terfenadine (a histamine H1 receptor antagonist [33]) and tubocurarine (a skeletal muscle relaxing alkaloid [34]) were obtained from Sigma Aldrich. All drug stocks were made using either DMSO or distilled water depending on solubility, then diluted using E3 embryo medium to make up the final concentrations for recording purposes with DMSO levels at 1%. ECG recordings were taken for 5 mins in 3 ml of embryo medium before addition of drug directly to the recording plate (1 ml) to produce the desired concentration by undergoing a 1 in 4 dilution.

### ECG data analysis

Analysis of the digitised ECG signal was carried out using LabChart and the mean intervals for the ECG parameters R-R, QRS, QT and QTc were calculated. For drug treatments the percentage change in the corrected QT interval (QTc) before and after drug administration was compared. One way ANOVA analysis was then performed to determine statistical significance between differences observed.

Z factor scores [35] were also calculated to determine the robustness of the ECG system as a screening tool, using the mean QTc percentage change before and after drug treatment for each individual fish. The values before drug treatment were standardised. The Z factor score was calculated using the following equation: Z  = 1– [3×(σp+σn)/(µp - µn)],

where µp and σp represent mean and SD after treatment, while µn and σn represent mean and SD before treatment. Z factor scores fall into the following range: 1 =  ideal, 0.5–1 =  excellent, 0–0.5 =  marginal, <0 =  poor. Z factor scores can never be more than 1, and if the score is below 0 there is too much overlap between the control and treatment groups to reliably make a distinction. The temperature coefficient Q_10_ [36] was calculated to determine the effect of temperature using the following equation: Q_10_ =  (R2–R1)^(10/T2–T1)^ where R1 =  heart rate at temperature T1, R2 =  heart rate at temperature T2.

## Results

### ECG Signal Morphology and Reproducibility

**Figure 1:**
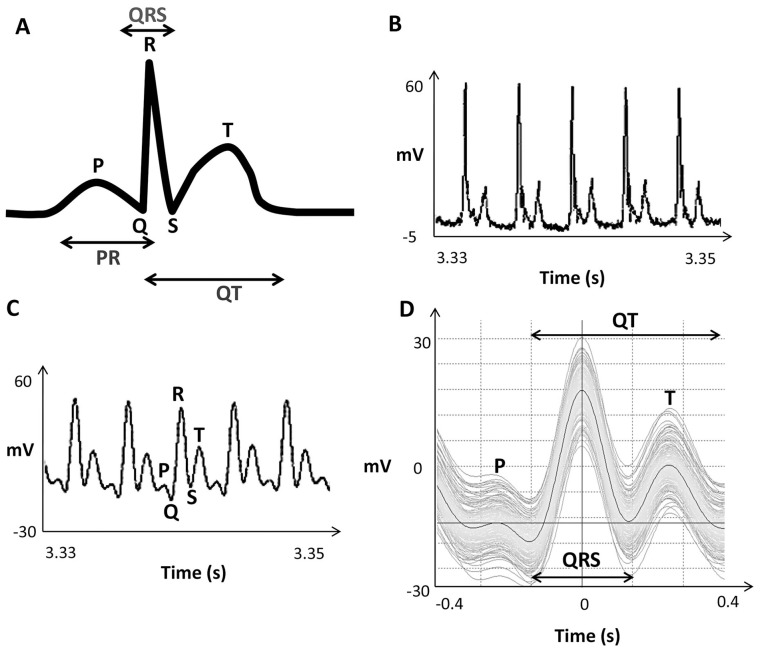
Detection of electrocardiographic signal. A) Depiction of an electrocardiogram and associated components, created using Adobe Photoshop CS6, B) 2 second section of a raw ECG recording from a larval zebrafish (3 dpf) without digital filtering, C) digitally filtered version of the ECG recording using low pass filtering to reduce background noise and produce a waveform suitable for analysis, D) analysed trace showing waveform reproducibility and stability during the recording period, with each line representing one cardiac cycle. The dark line in the middle represents the mean of all cycles from a 1 minute record.

The electrocardiogram represents summed electrical activity of the heart and consists of three main components, which starts with the small P wave (atrial depolarisation), the large QRS complex (ventricular depolarisation) and ends with the T wave (ventricular repolarisation) (Fig. 1A). To test the reliability of ECG measurements with a glass electrode based setup, as described previously [20], we used 3 dpf larvae as the heart is fully developed at this stage and because they are easier to manipulate in terms of positioning within wells of an agar plate following anaesthetisation, as it was more difficult to position older fish due to development of the swim bladder. After positioning of the electrode on the boundary between the atrium and ventricle, LabChart software was used to detect the ECG signal (Fig. 1B). Filtering steps were introduced to remove background noise and obtain a signal suitable for analysis (Fig. 1C) with distinctive P, QRS and T waves. The signal was found to be stable (no change in waveform) over the recording period (Fig. 1D), with all individual cycles being close to the average over the whole recording period. Components of the ECG waveform were analysed using the LabChart software, the settings being first adjusted to detect R waves. This was done by manually measuring the distance from one R wave to the next and inputting this into the settings. After the R waves were correctly identified the software could process the signal to produce an averaged waveform and calculate interval durations for different ECG components. Using this approach, measurement of particular ECG parameters (e.g. QT interval) could be performed automatically, allowing comparisons to be made between individual fish and those exposed to different environments or drug treatments.

### Detection of Effect of Motion Artefacts on ECG Signal

Although the above pilot experiments demonstrated reproducible detection of small amplitude P and T waves, there remained the possibility of motion artefact (variations in isoelectric point caused by heart movement). To check whether motion of the heart or the body muscle could affect ECG signals we compared signals recorded from the body surface over the heart using conditions where motion artefacts were excluded, using two independent analyses.

**Figure 2:**
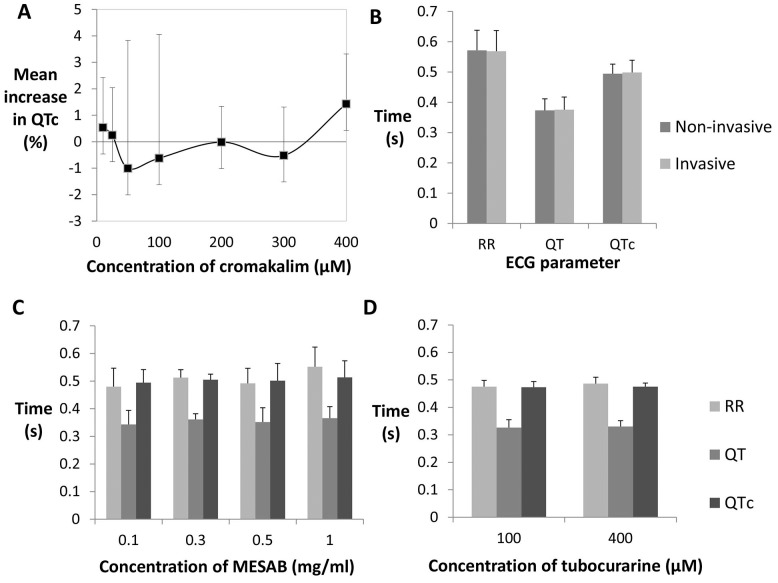
Exclusion of motion artefact and signal contamination. A) Mean ECG interval measurements obtained from 3 dpf larvae following motion artefact exclusion using cromakalim drug treatment (n = 5 per concentration), B) parallel invasive and non-invasive recordings (n = 4), C) prior MESAB anaesthesia for 5–10 minutes (n = 10 per concentration), and D) continuous tubocurarine exposure (n = 10 per concentration). Abbreviations: RR = time between the peak of one QRS complex to the next, QT = time of ventricular depolarisation and repolarisation, QTc = QT corrected for heart rate.

Firstly, recordings were performed with and without cromakalim and secondly, comparing invasive (intracardial) and non-invasive surface recordings. Cromakalim was added to the embryo holding chamber and ECG signals were recorded before and after drug exposure (QTc values shown in Table S1). As shown in Fig. 2A cromakalim caused no perturbation to the QTc interval in 3 dpf zebrafish (n = 5 per concentration), but also did not reduce contraction of cardiomyocytes at any of the tested concentrations. Additionally, excitation-contraction uncouplers 2,3-butanedione-monoxime (BDM) and blebbistatin were tested which were both found to completely remove myocardial contractility and still enable ECG signals to be recorded. However, both agents were also found to have adverse effects on the ECG and embryo survival (Fig. S1, S2 and S3, Movie S1). Therefore, as an independent approach we compared ECG signals from invasive and non-invasive electrode positioning. There was minimal difference between recordings taken invasively and non-invasively (n = 5; Fig. 2B, Table S2). Taken together, these results suggest that surface ECG recordings are a true representation of the electrical activity of the heart in 3 dpf zebrafish larvae.

We then investigated possible effects of various immobilising agents. The commonly used anaesthetic MESAB [37] and the paralytic agent tubocurarine [38] were tested. MESAB was administered for 5–10 minutes before transfer of the fish to the recording plate and tubocurarine was added to the recording plate for continuous exposure. Both methods had no specific effect (n = 10 per concentration) and produced comparable recordings (Fig. 2C and 2D). At high concentrations (1 mg/ml) MESAB led to increase of the R-R interval (*P*<0.05, see Table S3). Therefore, for ECG recording purposes 0.3 mg/ml of MESAB was subsequently used. With tubocurarine, immobilisation was only effective if the embryo was continuously exposed, limiting its suitability for treatment studies due to the possibility of drug interactions.

### Characterisation of ECG variables

**Figure 3:**
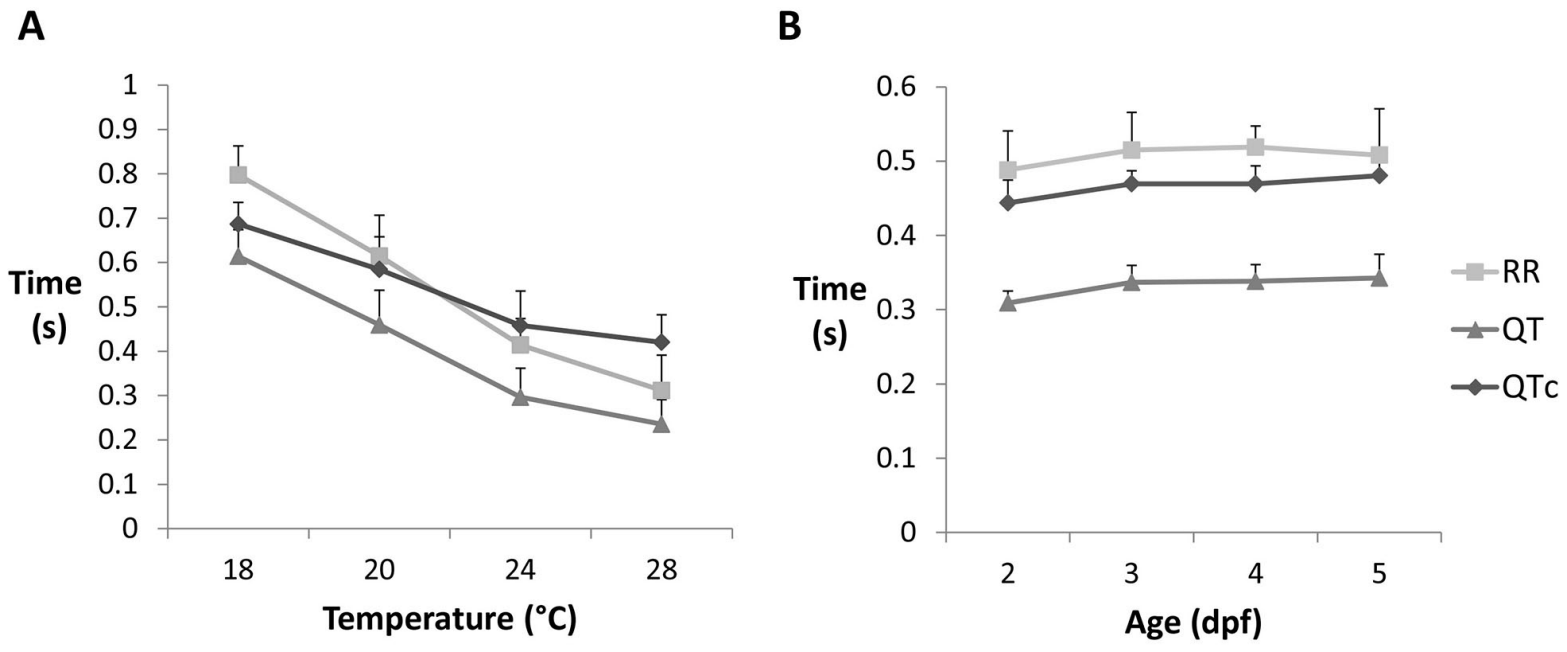
Optimisation of ECG recording system. A) Mean ECG interval measurements obtained from 3 dpf larvae following exposure to different ambient temperatures (n = 10), and B) over different developmental periods (2, 3, 4 and 5 days; n = 8).

To optimise the ECG recording method we assayed the effects of varying parameters, which may influence heart function or ECG detection. In poikilotherm fish species myocyte activity is reduced at lower temperatures, which can both decrease QRS amplitude and also lead to a reduction in heart rate (ƒH) as a natural adaptive mechanism to aid survival during colder climates or seasons [39]. At higher temperatures, increased ƒH facilitates greater cardiac output to support a higher metabolic activity/demand for oxygen consistent with normal biological rate function. Thus, temperature is expected to have an effect on heart rate and ECG measurements. Therefore, we tested the effect of temperature between 18 to 28 C^o^ in which range 3 dpf zebrafish larvae develop normally. Increasing ambient temperature led to increased mean heart rate and consequently decreased R-R, QT and QTc intervals (Fig. 3A, Table S4) demonstrating expected thermal sensitivity of heart function (n = 10, all *P*<0.05), confirmed by calculated Q_10_ coefficients of 2.7 over the 10°C range (Table S5).

Next we checked the developmental stages where reliable ECG signal could be detected (Fig. 3B). Reproducible ECGs could be recorded from zebrafish as early as long pec stage (2 dpf). ECG recordings taken from larvae at different stages (n = 8 per stage) showed differences in QT intervals when compared between 2 and 3, 2 and 4 and 2 and 5 dpf (all *P*<0.05), but not between 3 days and older (Table S6). Differences were seen in the QTc interval only between 2 and 5 dpf (*P*<0.05), while R-R intervals did not differ among age groups. Therefore, embryos over 2 days of age were used in subsequent experiments. For drug treatments, 3 dpf (protruding mouth stage) were chosen due to easier manipulation in wells of the recording plate (for reasons given above) and because they can start swallowing at this stage, aiding drug uptake.

**Figure 4:**
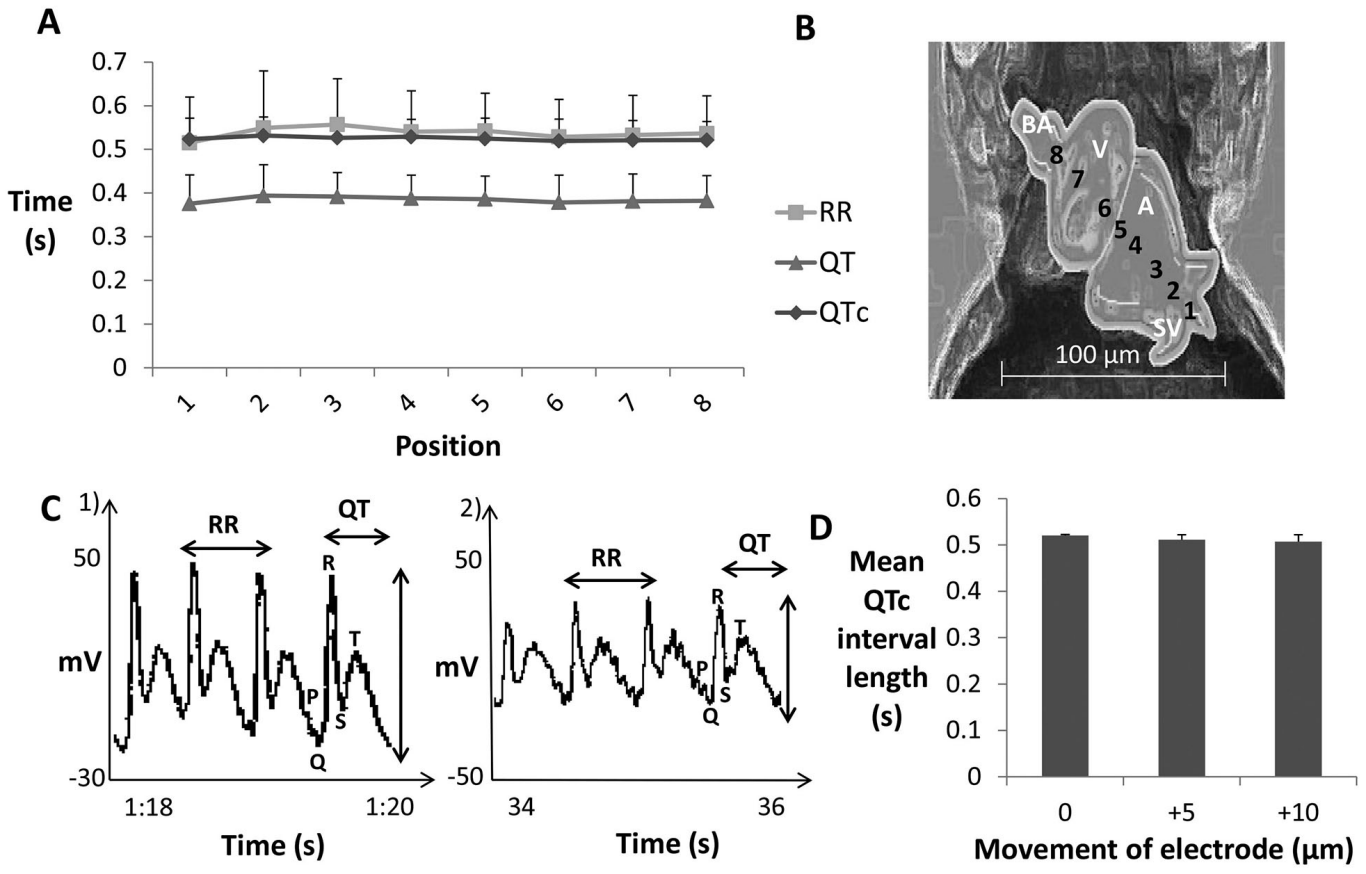
Investigation of effect of electrode position on ECG waveform. A) Mean ECG interval measurements obtained from 3 dpf larvae at different electrode positions over the heart (n = 10 per position), B) diagram of a 3 dpf larva with numbers identifying the different measurement positions; image created using Adobe Photoshop CS6, C) 1) 2 second raw ECG recording and 2) 2 second raw ECG recording from a 5 dpf larva after moving positive electrode 10 µm forward longitudinally, D) comparison of QTc intervals measured from 5 dpf larvae (n = 3) at starting position (atrium/ventricle boundary) and after movement of electrode longitudinally by 5 µm and 10 µm. Key: 1 =  sinus venosus, 2 =  between atrium and sinus venosus, 3 =  base of atrium, 4 =  apex of atrium, 5 =  between atrium and ventricle, 6 =  base of ventricle, 7 =  apex of ventricle and 8 =  bulbus arteriosus.

Differences in electrode placement in adult zebrafish ECGs was thought to be the cause of variation in T wave morphology and amplitude between different fish [2] [19]. For initial experiments, the electrode was always positioned on the atrium-ventricle boundary (see Fig. S4), as it was thought not to cause disturbance to cardiac ion channels [20]. To determine whether positioning of the electrode over the surface of the heart would impact on ECG measurement in larvae (n = 10), 8 different positions along the heart axis were tested (Fig. 4A and 4B, Table S7).

Movement of the positive electrode over the 5 dpf zebrafish heart by 10 microns longitudinally influenced the ECG waveform, particularly the amplitude (n = 3). However, the R-R, QT and QTc intervals did not change significantly (Fig. 4C and 4D, Table S8). Thus, electrode position was not critical for reliable ECG measurement, as the waveform morphology and component duration were unaffected.

### Detection of changes in ion channel function

Next we asked whether the ECG method developed on pre-feeding stage larvae could be used to detect the effect of QT prolonging drugs, and demonstrate its suitability for analysing cardiac dysfunction.

**Figure 5:**
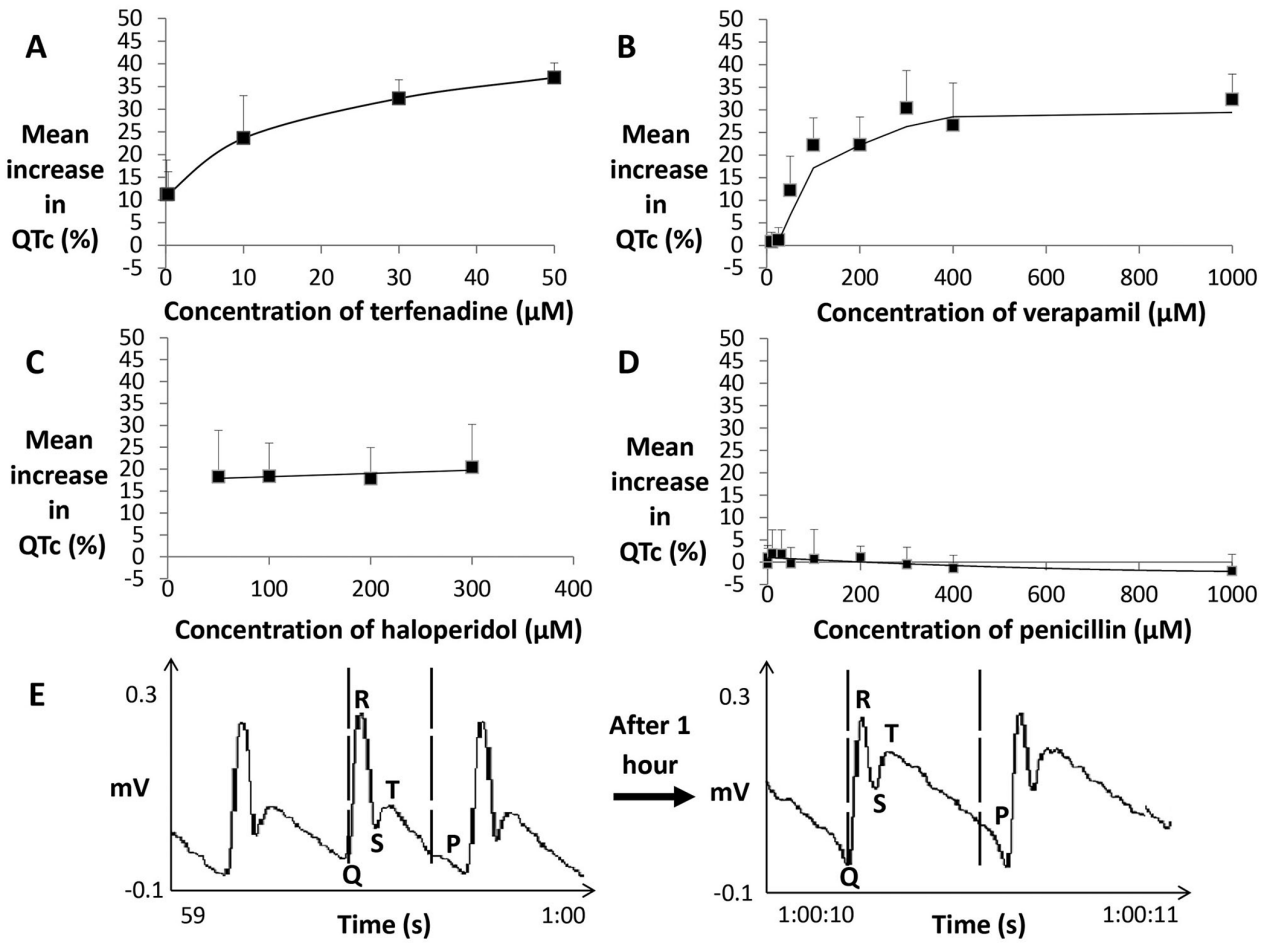
Effect of QT prolonging drugs on ECG measurements. A) Mean percentage change in QTc interval durations in 3 dpf larvae following treatment with terfenadine (n = 10 per concentration), B) verapamil (n = 10 per concentration), C) haloperidol (n = 10 per concentration) and D) penicillin (n = 10 per concentration). E) A raw ECG recording of a 3 dpf zebrafish before and 1 hour after treatment with 1 mM verapamil.

Several compounds can predispose to cardiac tachycardia due to QT prolonging effects caused by hERG channel blockade [40], the main cardiac ion channel involved in ventricular repolarisation. Haloperidol binds hERG channels more potently than other cardiac potassium channels, whereas terfenadine also has the potential to block other ion channels [33]. Quantitative comparison of the ECG waveform before and during terfenadine treatment revealed an increase in the QTc interval in a concentration-dependent manner (n = 8 per concentration, *P*<0.05; Fig. 5A, Table S9). Apparent QT prolongation was observed with widening of the T wave (see Fig. S5). QT prolongation with haloperidol has been previously reported in adult zebrafish [2]. In contrast to terfenadine, haloperidol led to an apparent QT prolongation, which was found to be similar at various concentrations (n = 8 per concentration, *P*<0.05; Fig. 5B, Table S10). This was also seen with pimozide (n = 8 per concentration, Fig. S6, Table S11). Verapamil (Fig. 5C), a known hERG blocker *in vitro* also increased the QTc interval (n = 8 per concentration, *P*<0.05; Fig. 5E, Table S12). Penicillin treatment was used as a negative control as it has been shown not to cause QT prolongation in adult zebrafish [2], and it was found to have no effect on the QTc interval in zebrafish larvae (n = 8 per concentration, Fig. 5D, Table S13), confirming high specificity of our system to detect compounds that have an apparent QT prolonging effect. Taken together, our results are in general agreement with those obtained in adult zebrafish and human studies [2], confirming the drug specificity for apparent QT prolongation in zebrafish larvae.

Additionally, with terfenadine the development of an arrhythmia was also observed, which was time and concentration-dependent causing marked alterations to the waveform (see Fig. S7). Terfenadine, haloperidol and pimozide also induced an atrioventricular (AV) block at particular concentrations and exposure times (Movies S2, S3 and S4). Thus, the ECG measurement system is also able to detect changes other than QT prolongation, making it useful as a readout for other cardiotoxic conditions.

Finally, we asked if the ECG detection system was suitable as a drug toxicity screening tool. Therefore we calculated Z-factor scores of reproducibility in repeat experiments [41] (Table 1). Z-factor scores for terfenadine at 30 and 50 µM were calculated as 0.68 and 0.78 respectively, which fall into the excellent range (>0.5). Additionally, for controls (e.g. low drug concentration, solvent or no drug) the Z-factor was well outside the range (<0). For verapamil at 25 µM, penicillin at 25 µM, no drug control and DMSO solvent control the Z factors were 6.75, −6.56, −6.67–2.71, respectively. These results demonstrate that the ECG measurement system is sensitive in reproducibly detecting changes caused by high concentrations of QT prolonging drugs as compared to various controls and argue for its utility as a drug toxicity screening tool.

**Table 1:** Z-factor scores for different treatment regimes.

Treatment	Mean Z-factor score
Terfenadine 0.1 µM	−1.671
Terfenadine 0.3 µM	−0.254
Terfenadine 10 µM	−0.371
Terfenadine 30 µM	0.676
Terfenadine 50 µM	0.784
Penicillin 25 µM	−6.562
Verapamil 25 µM	−6.747
DMSO (0.1%)	−2.708
No drug	−6.673

## Discussion

### Optimisation of ECG Recording

We have optimised the parameters of an ECG recording system to measure electrocardiograms from zebrafish embryos and larvae (ranging from 2 to 5 days), using a similar technique to that previously developed for 5 dpf embryos [20]. Compared to earlier studies, where ECG recordings detected only P and R waves, with our system all components of the ECG could be observed. In adult zebrafish, the primary pacemaking site originates at the sinus venosus-atrial junction and propagates from there during atrial diastole into the ventricle [29]. In 3 dpf zebrafish the primary pacemaking site is situated in the dorsal right quadrant of the sinoatrial ring [42]. The pacemaker site is identified by a molecular marker islet-1, whose expression is required for pacemaker activity [43]. Hence, as early as 2 dpf all the elements required to generate compound action potentials and effective cardiac conduction are present, enabling the measurement of electrocardiograms.

When optimising the ECG recording procedure, tubocurarine was only found to be effective as a sedating agent if added to the recording plate directly, so that embryos were continuously exposed. However, it did not affect ƒH at any of the tested concentrations, whereas MESAB induced bradycardia at very high concentrations. To eliminate motion artefacts, cromakalim treatment was tested as it has been previously used as an excitation-contraction uncoupler [44], causing membrane hyperpolarisation. However, in zebrafish embryos it had no effect on the ECG or heart contractility, which agrees with other studies [9]. Additionally, BDM and blebbistatin were also tested; both were found to block myocardial contractility, as previously documented [45], but also had adverse effects on the ECG signals that were recorded and embryo survival. Therefore, to counteract these limitations we also used intracardial ECG recording to demonstrate there was no difference between invasive and non-invasive recordings, and hence the latter could be used for assessing drug treatments.

### A zebrafish embryo model for QT prolongation

Terfenadine causes QT prolongation in adult zebrafish [2], which we also demonstrate in zebrafish embryos. The mean QTc duration was dose and time-dependent, with lower concentrations giving smaller changes and requiring longer incubation times. Additionally, the dose-response was broadly sigmoidal, similar to an ECG study with astemizole in adult zebrafish [2]. Haloperidol also caused QT prolongation in zebrafish embryos, although at higher concentrations than terfenadine and the effect was not dose-dependent. Changes in the ECG waveform following exposure to both QT prolonging drugs were similar to those observed in adult zebrafish, i.e. widening of the QRS complex and T wave [2].

One surprising result was that verapamil also caused QT prolongation, in contrast to the response in mammals [46], but consistent with data obtained in *in vitro* hERG cell assays (e.g. human embryonic kidney cells) [47] and *Xenopus laevis* oocytes [48], where verapamil is a potent hERG blocker. Verapamil specifically binds to the alpha 1 c subunit of the L-type calcium channel, preventing it from opening and therefore reducing myocardial contractility by decreasing calcium influx [14].

At concentrations above 1 mM verapamil caused A/V decoupling of the zebrafish ECG (data not shown), consistent with other studies [9]. This is similar to the mammalian response where verapamil injections may cause arrhythmias, a common side effect of anti-arrhythmics in overdose conditions [49]. Cardiotoxicity studies with zebrafish embryos to detect hERG blockade only reported bradycardia following verapamil exposure [8], although younger (48 hpf) embryos were used and the concentrations may have been too low to detect any other effect. As blockade of the IKs channel (other main potassium channel affecting cardiac repolarisation) is also linked with prolongation of the QT interval [50] and verapamil is an IKs channel inhibitor [51] this may have contributed to the QT prolongation observed, as effective concentrations were relatively high. However, further studies are required to identify the exact mechanism of action of verapamil on zebrafish cardiac ion channels.

### A zebrafish embryo model for cardiotoxicity testing

An additional effect of terfenadine and haloperidol was a 2∶1 AV block that was consistent for each fish tested but dependent on both dose and exposure time. This type of AV block displayed many features similar to those seen in humans (Movies S2, S3, S4). It is known that when zERG is inhibited, a 2∶1 AV block is observed [52], as seen in similar experiments with mammals [53]. Currently, there is no evidence for 2∶1 AV block as a result of blockage of other ion channels [8]. While in humans AV block only occurs rarely, it occurs in newborns and neonates alongside QT prolongation [52]. Similarly, in zebrafish embryos QT prolongation occurred before, and in some cases alongside 2∶1 AV block. Although in humans the QT prolonging drug astemizole causes 2∶1 AV block at very high concentrations, this occurs at lower doses in zebrafish embryos [52], suggesting that zERG is more sensitive than the hERG channel. Alternatively, the zebrafish ventricle may rely more upon the IKr current for repolarisation than the atrium and therefore is more vulnerable to AV block and also arrhythmia development, which was also observed with terfenadine characterised by ectopic beats and irregularities in heart rhythm [52].

The zebrafish embryo/larva is an attractive model for studying cardiotoxicity due to its similarity to humans in terms of cardiac physiology, as well as its easy accessibility, fast reproduction, high fecundity, short generation times compared to other animal models and its contribution to the 3 Rs (replacement, refinement and reduction) of animals in research. Currently, QT interval measurements in other animals show very wide variation and the effects seen are often small [52]. While the readouts currently obtainable with zebrafish (bradycardia, QT prolongation, 2∶1 AV block, arrhythmias) may be good surrogates for cardiac dysfunction in humans [52], zebrafish embryos may be more sensitive to QT prolongation drugs compared to mammalian systems. It could therefore be useful to test the QT-prolonging potential of drugs currently under development using ECG technology that is non-invasive, utilises live animals and is robust enough to use as a screening tool based on calculated z factor scores which show that the system is able to differentiate between natural variation within samples and specific changes caused by drugs.

Although much recent research effort has focused on developing high throughput assays to assess QT prolonging compounds using transgenic zebrafish [8], the information that can be obtained from ECG measurements is more insightful, as it can provide detail of electrophysiological dynamics such as the possible mechanisms underlying arrhythmogenesis. Besides compound screening, the zebrafish embryo ECG system also offers the opportunity to study cardiac mutants and associated pathologies. The feasibility of mutagenesis screens allowed identification of a variety of cardiac-specific mutants affecting both heart development and physiology [54] [55]. One major drawback with using zebrafish for compound screening is that drug penetration and metabolism can be unpredictable. For example, it was recently found that in zebrafish larvae terfenadine is almost completely metabolised to azacyclone and terfenadine alcohol [56], while in humans fexofenadine is predominantly formed. This suggests that drug metabolism in zebrafish may utilise a different pathway. On the other hand, metabolites of verapamil were identified in zebrafish larvae that were the same as those produced in humans [56]. Therefore, additional studies in this area would be necessary to further validate the zebrafish as an additional animal model for preclinical testing in cardiotoxicity screens [28].

## Supporting Information

Figure S1 Exclusion of motion artefact with BDM. Screenshots from a 3 dpf larval zebrafish ECG recording before (A) and after 2 minutes (B) of treatment with 15 mM BDM.
(TIF)

Figure S2 Effect on QTc with excitation-contraction uncoupler BDM. Mean QTc interval durations of 3 dpf zebrafish larvae before and after 2 minutes of treatment with 15 mM BDM (n = 10, *P*>0.05).
(TIF)

Figure S3 Effect on QTc with excitation-contraction uncoupler blebbistatin. Mean QTc interval durations of 3 dpf zebrafish larvae before and after 10 minutes of treatment with 15 µM blebbistatin (n = 5, *P*<0.05).
(TIF)

Figure S4 Electrode positioning for ECG recording. Depiction of a 2 µM electrode tip positioned on the heart of a 60 hpf zebrafish embryo to illustrate the relative sizes.
(TIF)

Figure S5 QT prolongation with terfenadine. Processed waveform showing QT prolongation with 50 µM terfenadine in a 3 dpf larva: A) Averaged ECG waveform (red line) before drug administration, B) T wave shift to the right observed after 1 minute of terfenadine treatment, C) more pronounced QT prolongation after 7 minutes.
(TIF)

Figure S6 QT prolongation with pimozide. The QT prolonging drug pimozide was found to cause a statistically significant increase in the corrected QT interval in 3 dpf zebrafish (n = 8, *P<*0.05).
(TIF)

Figure S7 Development of arrhythmia with terfenadine. ECG screenshot of : A) 3 dpf zebrafish larva before treatment and B) after 40 minutes of treatment with 50 µM terfenadine showing an arrhythmic phenotype with the presence of ectopic beats.
(TIF)

Table S1 Effect of cromakalim on QTc interval duration.
(DOCX)

Table S2 Measured ECG intervals from invasive and non-invasive recordings.
(DOCX)

Table S3 Measured ECG intervals following MESAB and tubocurarine treatments.
(DOCX)

Table S4 Effect of temperature on ECG intervals.
(DOCX)

Table S5 Calculated Q10 coefficients for different temperature ranges.
(DOCX)

Table S6 ECG intervals measured at different stages.
(DOCX)

Table S7 Measured QTc intervals at different positions on the heart.
(DOCX)

Table S8 Measured QTc intervals following movement of electrode.
(DOCX)

Table S9 Effect of terfenadine on QTc interval duration.
(DOCX)

Table S10 Effect of haloperidol on QTc interval duration.
(DOCX)

Table S11 Effect of pimozide on QTc interval duration.
(DOCX)

Table S12 Effect of verapamil on QTc interval duration.
(DOCX)

Table S13 Effect of penicillin on QTc interval duration.
(DOCX)

Movie S1 Reduction of myocardial contractility with blebbistatin. 3 dpf zebrafish larva before and after 20 minutes of treatment with 15 µM blebbistatin.
(WMV)

Movie S2 AV block with terfenadine. 3 dpf zebrafish larva before and after 30 minutes of treatment with 50 µM terfenadine.
(WMV)

Movie S3 AV block with haloperidol. 3 dpf zebrafish larva before and after 45 minutes of treatment with 50 µM haloperidol.
(WMV)

Movie S4 AV block with pimozide. 3 dpf zebrafish larva before and after 20 minutes of treatment with 100 µM pimozide.
(WMV)
