# Supplementary figures and images for: Optimisation of Embryonic and Larval ECG Measurement in Zebrafish for Quantifying the Effect of QT Prolonging Drugs
Source: PLoS One. 2013 Apr 8;8(4):e60552. doi: 10.1371/journal.pone.0060552 (PMC3620317; doi:10.1371/journal.pone.0060552)

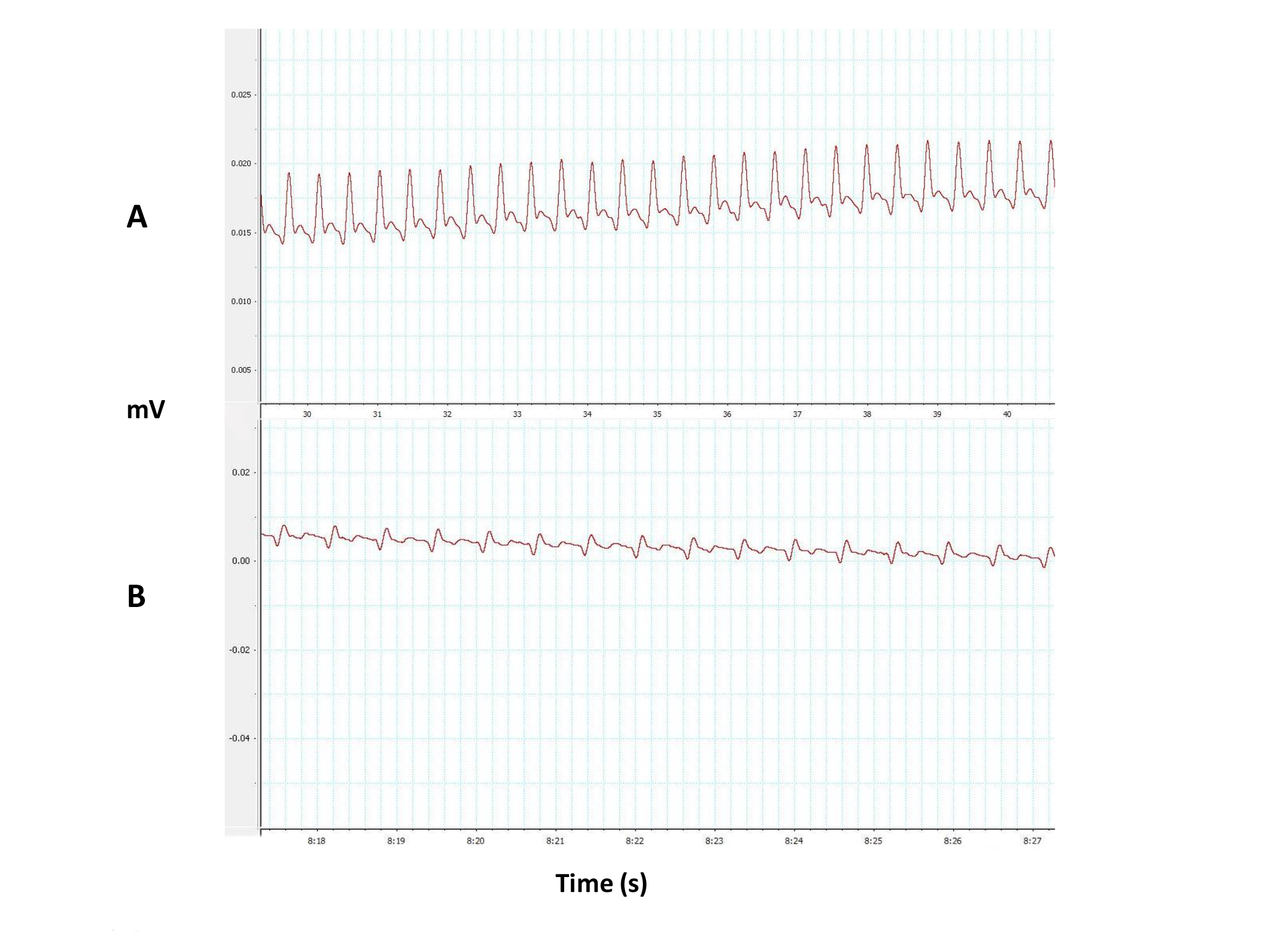

Supplement: Figure S1 — Exclusion of motion artefact with BDM. Screenshots from a 3 dpf larval zebrafish ECG recording before (A) and after 2 minutes (B) of treatment with 15 mM BDM. (TIF) [file pone.0060552.s001.tif]

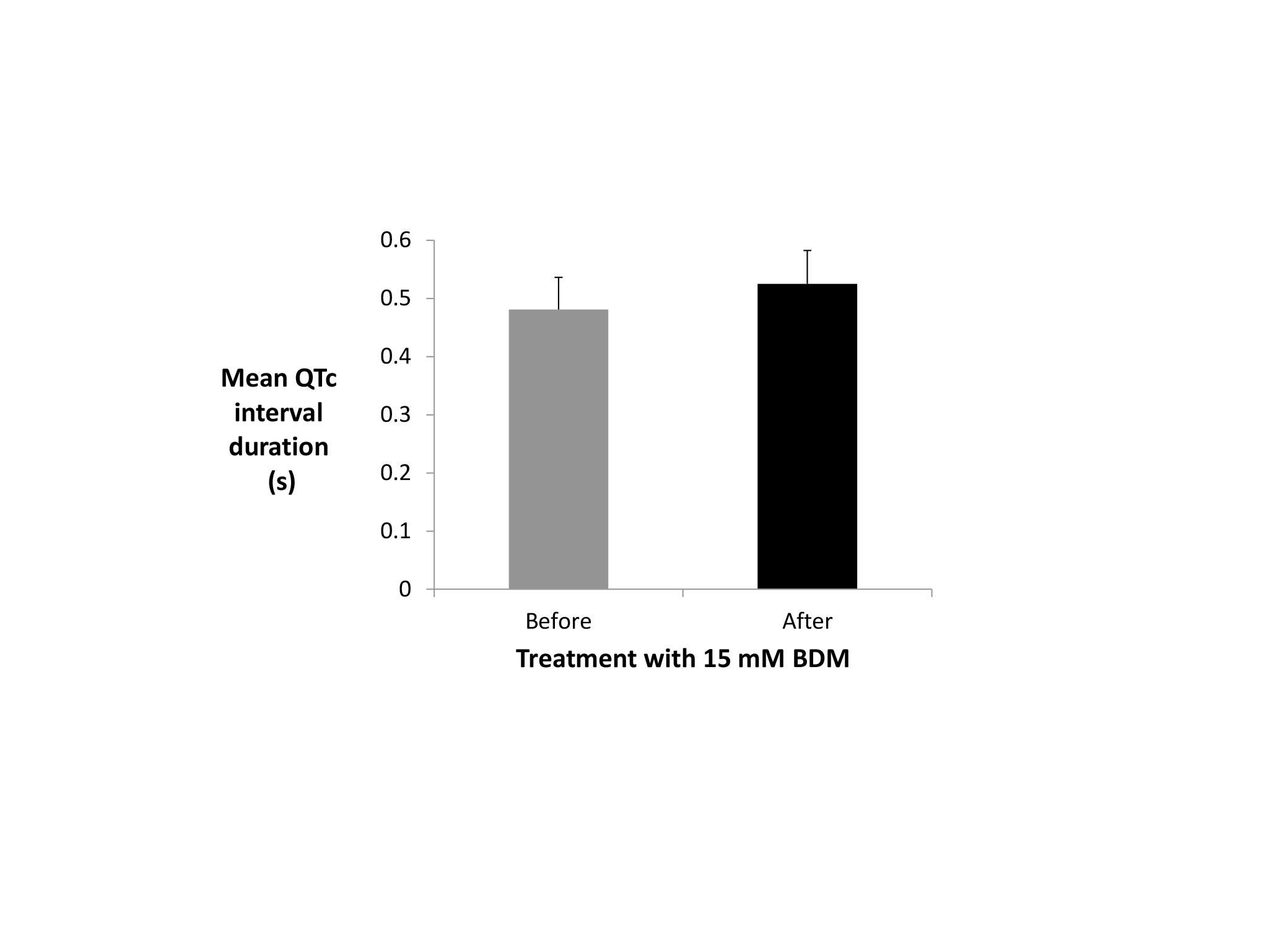

Supplement: Figure S2 — Effect on QTc with excitation-contraction uncoupler BDM. Mean QTc interval durations of 3 dpf zebrafish larvae before and after 2 minutes of treatment with 15 mM BDM (n = 10, P>0.05). (TIF) [file pone.0060552.s002.tif]

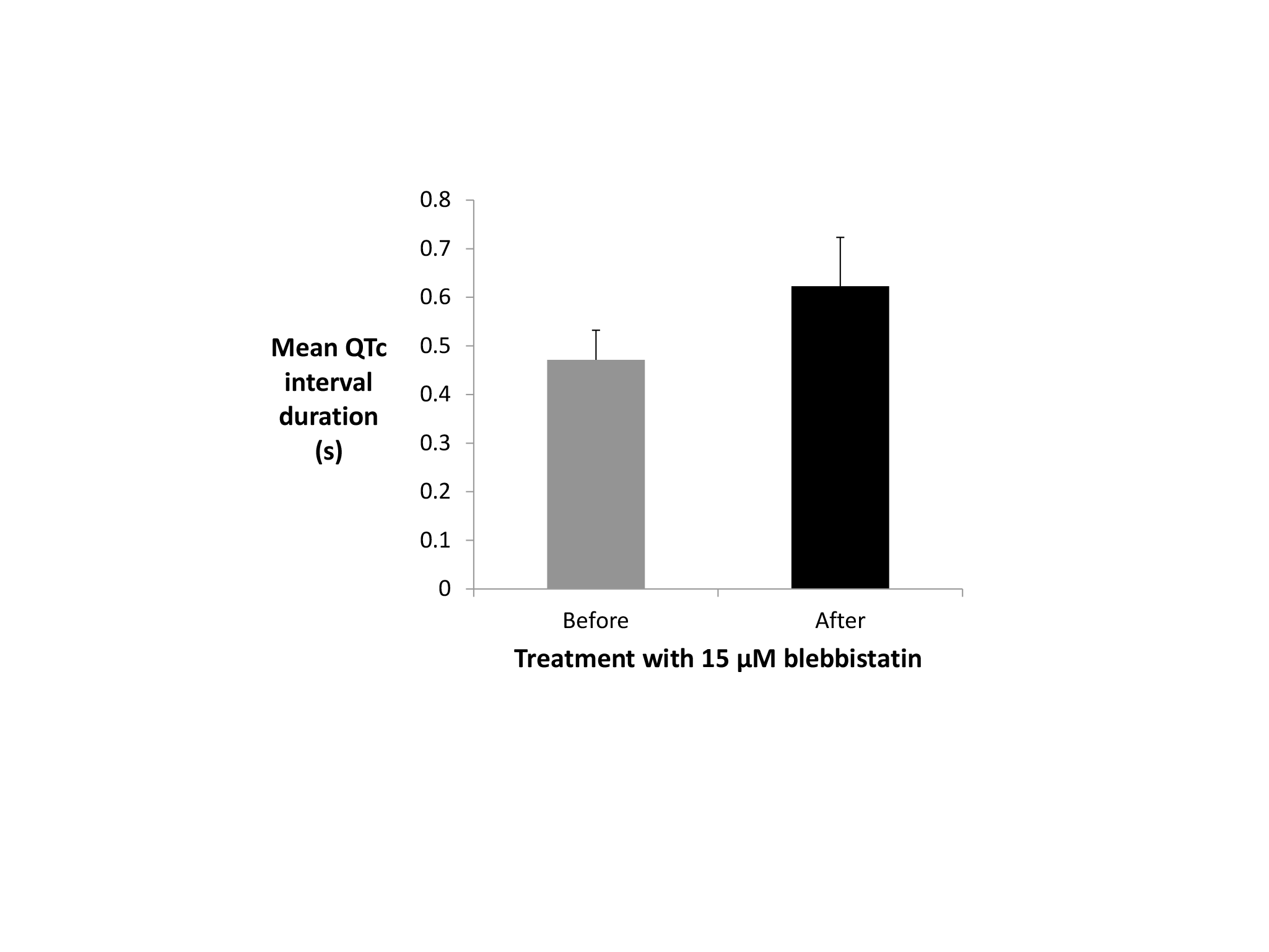

Supplement: Figure S3 — Effect on QTc with excitation-contraction uncoupler blebbistatin. Mean QTc interval durations of 3 dpf zebrafish larvae before and after 10 minutes of treatment with 15 µM blebbistatin (n = 5, P<0.05). (TIF) [file pone.0060552.s003.tif]

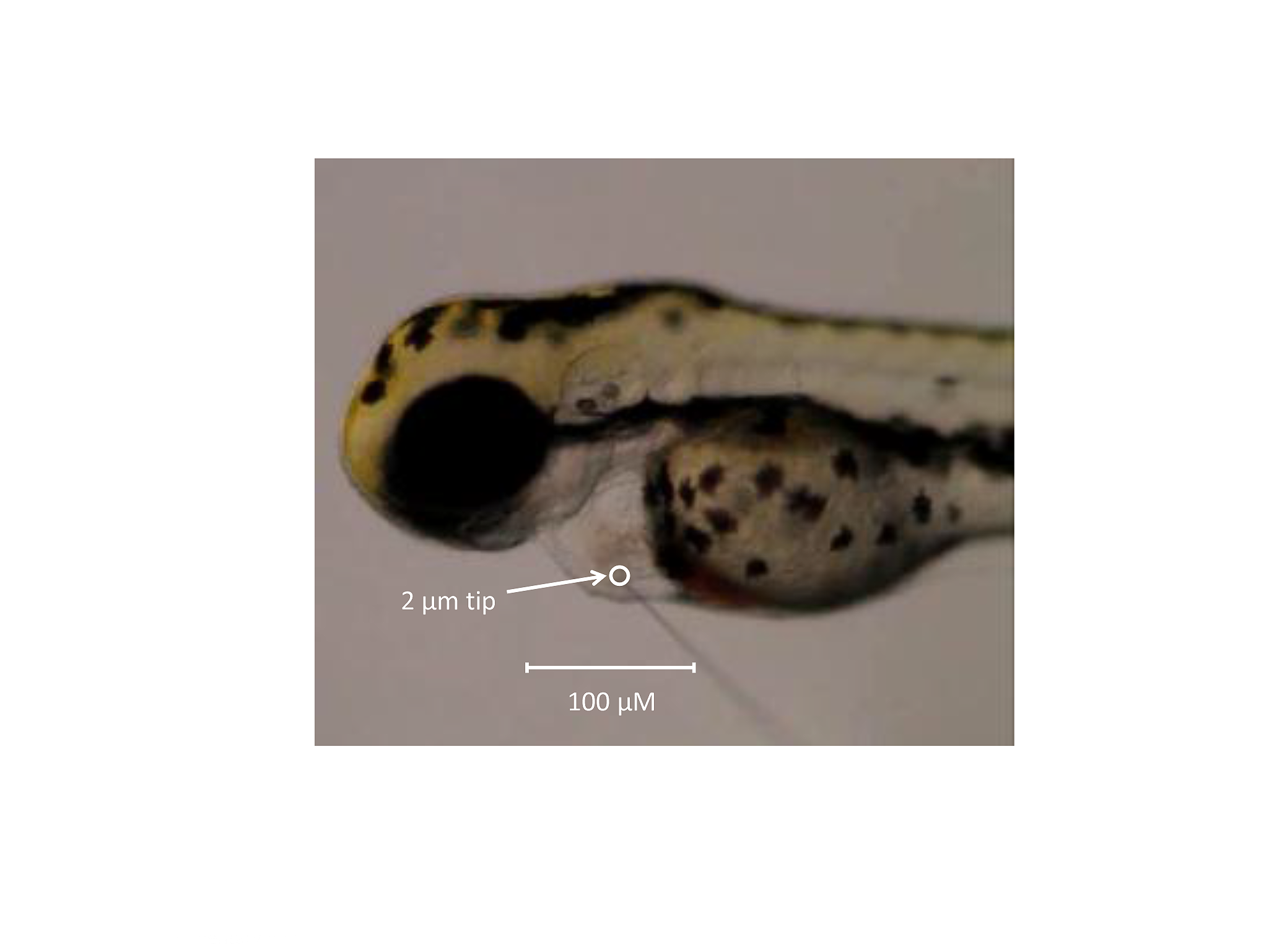

Supplement: Figure S4 — Electrode positioning for ECG recording. Depiction of a 2 µM electrode tip positioned on the heart of a 60 hpf zebrafish embryo to illustrate the relative sizes. (TIF) [file pone.0060552.s004.tif]

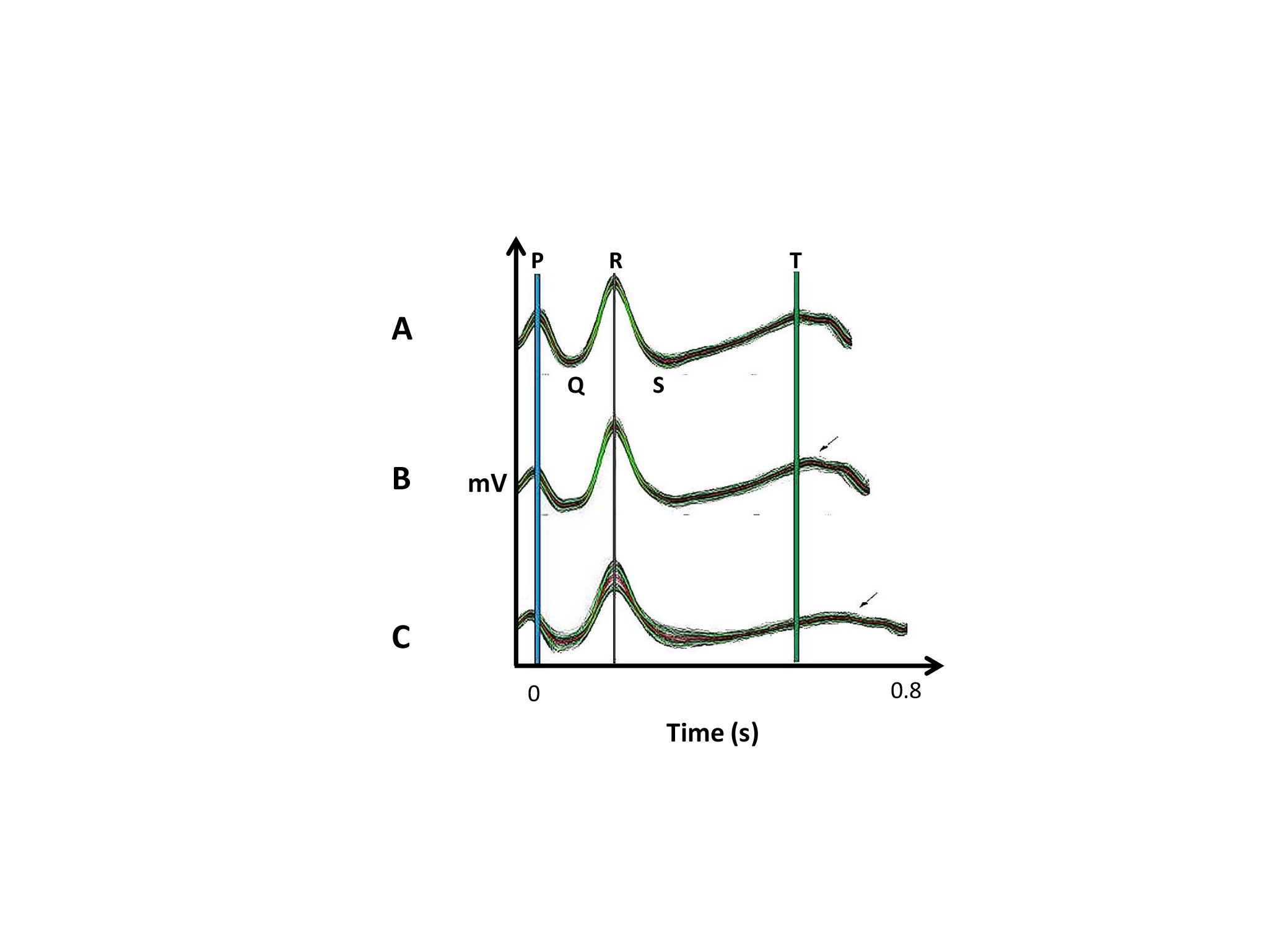

Supplement: Figure S5 — QT prolongation with terfenadine. Processed waveform showing QT prolongation with 50 µM terfenadine in a 3 dpf larva: A) Averaged ECG waveform (red line) before drug administration, B) T wave shift to the right observed after 1 minute of terfenadine treatment, C) more pronounced QT prolongation after 7 minutes. (TIF) [file pone.0060552.s005.tif]

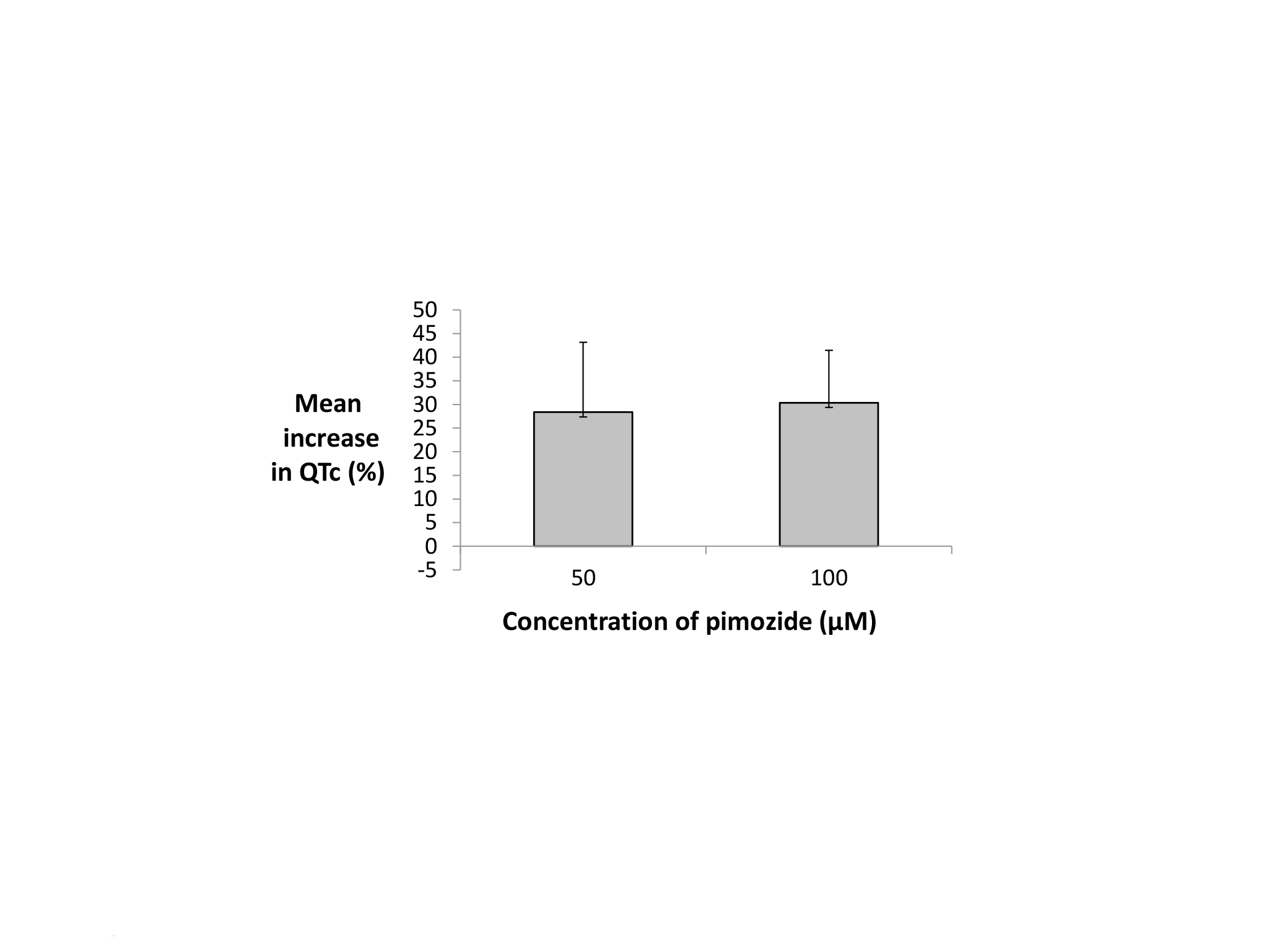

Supplement: Figure S6 — QT prolongation with pimozide. The QT prolonging drug pimozide was found to cause a statistically significant increase in the corrected QT interval in 3 dpf zebrafish (n = 8, P<0.05). (TIF) [file pone.0060552.s006.tif]

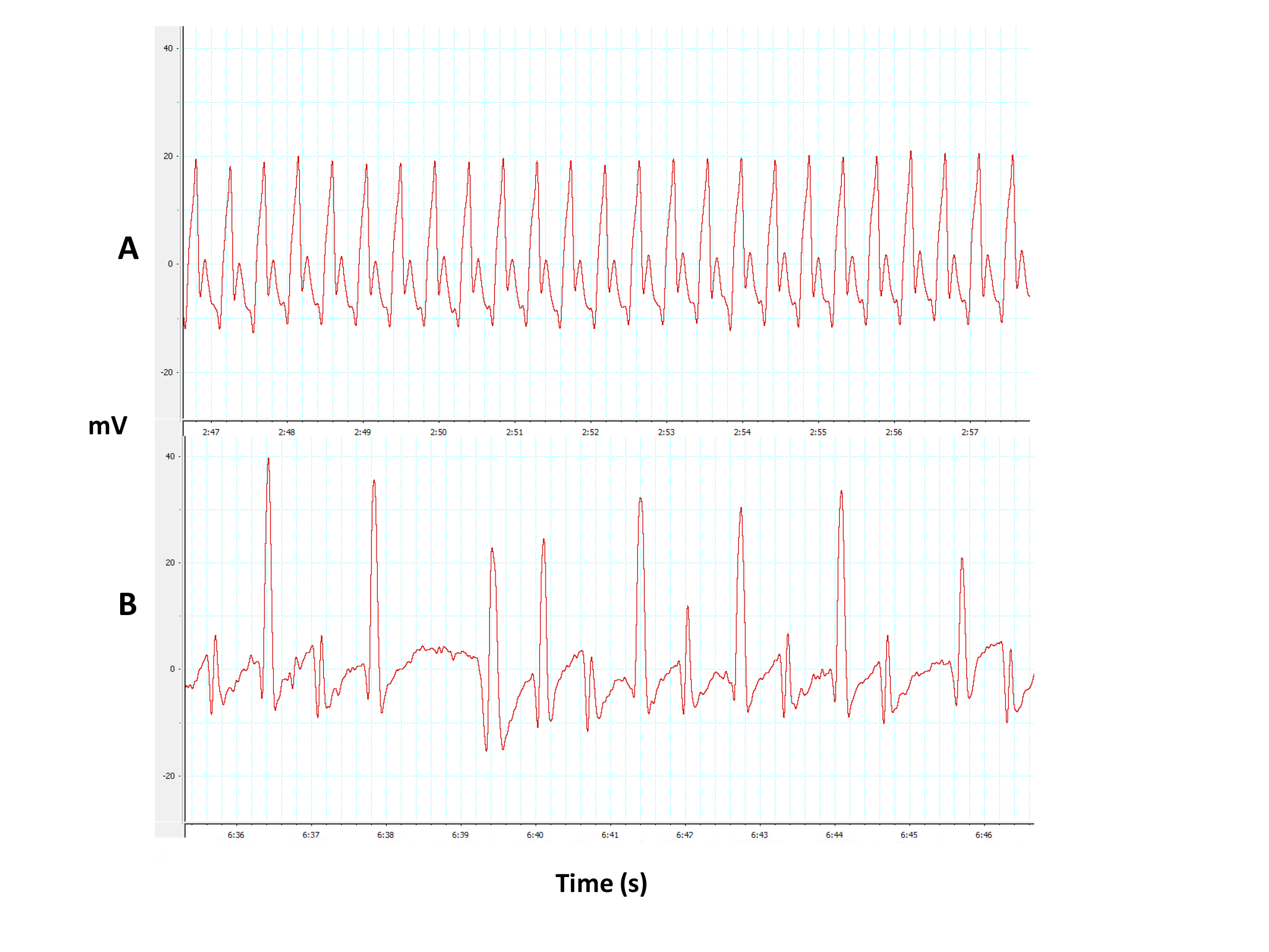

Supplement: Figure S7 — Development of arrhythmia with terfenadine. ECG screenshot of : A) 3 dpf zebrafish larva before treatment and B) after 40 minutes of treatment with 50 µM terfenadine showing an arrhythmic phenotype with the presence of ectopic beats. (TIF) [file pone.0060552.s007.tif]
