# Supplementary material for: Optimisation of Embryonic and Larval ECG Measurement in Zebrafish for Quantifying the Effect of QT Prolonging Drugs
Source: PLoS One. 2013 Apr 8;8(4):e60552. doi: 10.1371/journal.pone.0060552 (PMC3620317; doi:10.1371/journal.pone.0060552)
Supplement: Table S1 — Effect of cromakalim on QTc interval duration. (DOCX) [file pone.0060552.s008.docx]

| Concentration of cromakalim (µM) | Mean QTc interval duration (s) | |
| --- | --- | --- |
|  | Before | After |
| 10 | 0.458 | 0.461 |
| 25 | 0.459 | 0.460 |
| 50 | 0.469 | 0.464 |
| 100 | 0.453 | 0.449 |
| 200 | 0.476 | 0.476 |
| 300 | 0.475 | 0.472 |
| 400 | 0.468 | 0.474 |
| *n = 5 per concentration* | | |
