# Supplementary material for: Optimisation of Embryonic and Larval ECG Measurement in Zebrafish for Quantifying the Effect of QT Prolonging Drugs
Source: PLoS One. 2013 Apr 8;8(4):e60552. doi: 10.1371/journal.pone.0060552 (PMC3620317; doi:10.1371/journal.pone.0060552)
Supplement: Table S2 — Measured ECG intervals from invasive and non-invasive recordings. (DOCX) [file pone.0060552.s009.docx]

| Sample | Interval duration (s) | | | | | |
| --- | --- | --- | --- | --- | --- | --- |
|  | Invasive | Non-invasive | Invasive | Non-invasive | Invasive | Non-invasive |
|  | RR | RR | QT | QT | QTc | QTc |
| Larva 1 | 0.478 | 0.478 | 0.325 | 0.348 | 0.470 | 0.504 |
| Larva 2 | 0.595 | 0.604 | 0.370 | 0.351 | 0.479 | 0.451 |
| Larva 3 | 0.568 | 0.569 | 0.408 | 0.360 | 0.541 | 0.478 |
| Larva 4 | 0.634 | 0.636 | 0.401 | 0.435 | 0.504 | 0.546 |
| Larva 5 | 0.569 | 0.571 | 0.376 | 0.374 | 0.499 | 0.495 |
