# Supplementary material for: Optimisation of Embryonic and Larval ECG Measurement in Zebrafish for Quantifying the Effect of QT Prolonging Drugs
Source: PLoS One. 2013 Apr 8;8(4):e60552. doi: 10.1371/journal.pone.0060552 (PMC3620317; doi:10.1371/journal.pone.0060552)
Supplement: Table S3 — Measured ECG intervals following MESAB and tubocurarine treatments. (DOCX) [file pone.0060552.s010.docx]

| Concentration of MESAB (mg/ml) | Mean interval duration (s) | | |
| --- | --- | --- | --- |
|  | RR | QT | QTc |
| 0.1 | 0.47979 | 0.34285 | 0.49441 |
| 0.3 | 0.51232 | 0.36139 | 0.5049 |
| 0.5 | 0.49169 | 0.35149 | 0.50122 |
| 1 | 0.55168 | 0.36565 | 0.51327 |
| Concentration of +/- tubocurarine pentachloride (μM) | | | |
| 100 | 0.47555 | 0.32631 | 0.47325 |
| 400 | 0.48634 | 0.33055 | 0.4755 |
| *n = 10 per concentration* | | | |
