# Supplementary material for: Optimisation of Embryonic and Larval ECG Measurement in Zebrafish for Quantifying the Effect of QT Prolonging Drugs
Source: PLoS One. 2013 Apr 8;8(4):e60552. doi: 10.1371/journal.pone.0060552 (PMC3620317; doi:10.1371/journal.pone.0060552)
Supplement: Table S4 — Effect of temperature on ECG intervals. (DOCX) [file pone.0060552.s011.docx]

| Sample | Interval durations at different temperatures (s) | | | | | | | | | | | |
| --- | --- | --- | --- | --- | --- | --- | --- | --- | --- | --- | --- | --- |
|  | 18ºC | | | 20ºC | | | 24ºC | | | 28ºC | | |
|  | RR | QT | QTc | RR | QT | QTc | RR | QT | QTc | RR | QT | QTc |
| Larva 1 | 0.704 | 0.493 | 0.588 | 0.573 | 0.454 | 0.600 | 0.368 | 0.201 | 0.331 | 0.233 | 0.162 | 0.336 |
| Larva 2 | 0.857 | 0.615 | 0.664 | 0.772 | 0.562 | 0.640 | 0.457 | 0.355 | 0.526 | 0.317 | 0.281 | 0.498 |
| Larva 3 | 0.830 | 0.627 | 0.689 | 0.620 | 0.493 | 0.626 | 0.359 | 0.248 | 0.415 | 0.282 | 0.196 | 0.369 |
| Larva 4 | 0.873 | 0.635 | 0.680 | 0.717 | 0.505 | 0.596 | 0.547 | 0.362 | 0.489 | 0.518 | 0.341 | 0.474 |
| Larva 5 | 0.768 | 0.646 | 0.737 | 0.649 | 0.502 | 0.622 | 0.410 | 0.336 | 0.525 | 0.313 | 0.282 | 0.503 |
| Larva 6 | 0.877 | 0.721 | 0.770 | 0.483 | 0.440 | 0.633 | 0.455 | 0.400 | 0.590 | 0.341 | 0.277 | 0.474 |
| Larva 7 | 0.807 | 0.622 | 0.692 | 0.647 | 0.509 | 0.633 | 0.405 | 0.259 | 0.406 | 0.269 | 0.199 | 0.384 |
| Larva 8 | 0.742 | 0.598 | 0.695 | 0.642 | 0.471 | 0.588 | 0.395 | 0.269 | 0.429 | 0.302 | 0.210 | 0.383 |
| Larva 9 | 0.813 | 0.632 | 0.700 | 0.495 | 0.292 | 0.415 | 0.346 | 0.228 | 0.389 | 0.295 | 0.197 | 0.362 |
| Larva 10 | 0.706 | 0.550 | 0.654 | 0.551 | 0.365 | 0.492 | 0.399 | 0.304 | 0.481 | 0.249 | 0.210 | 0.422 |
