# Supplementary material for: Optimisation of Embryonic and Larval ECG Measurement in Zebrafish for Quantifying the Effect of QT Prolonging Drugs
Source: PLoS One. 2013 Apr 8;8(4):e60552. doi: 10.1371/journal.pone.0060552 (PMC3620317; doi:10.1371/journal.pone.0060552)
Supplement: Table S5 — Calculated Q10 coefficients for different temperature ranges. (DOCX) [file pone.0060552.s012.docx]

| Temperature (⁰C) | Q_10_ |
| --- | --- |
| 18-20 | 5.187963 |
| 18-24 | 3.165493 |
| 18-28 | 2.739147 |
| 20-24 | 2.820046 |
| 20-28 | 2.524591 |
| 24-28 | 2.340546 |
