# Supplementary material for: Optimisation of Embryonic and Larval ECG Measurement in Zebrafish for Quantifying the Effect of QT Prolonging Drugs
Source: PLoS One. 2013 Apr 8;8(4):e60552. doi: 10.1371/journal.pone.0060552 (PMC3620317; doi:10.1371/journal.pone.0060552)
Supplement: Table S6 — ECG intervals measured at different stages. (DOCX) [file pone.0060552.s013.docx]

| Age (dpf) | Mean interval duration (s) | | |
| --- | --- | --- | --- |
|  | RR | QT | QTc |
| 2 | 0.488 | 0.309 | 0.444 |
| 3 | 0.515 | 0.337 | 0.470 |
| 4 | 0.519 | 0.338 | 0.469 |
| 5 | 0.508 | 0.343 | 0.481 |
| *n = 8 per stage* | | | |
