# Supplementary material for: Optimisation of Embryonic and Larval ECG Measurement in Zebrafish for Quantifying the Effect of QT Prolonging Drugs
Source: PLoS One. 2013 Apr 8;8(4):e60552. doi: 10.1371/journal.pone.0060552 (PMC3620317; doi:10.1371/journal.pone.0060552)
Supplement: Table S7 — Measured QTc intervals at different positions on the heart. (DOCX) [file pone.0060552.s014.docx]

| Sample | Position | | | | | | | |
| --- | --- | --- | --- | --- | --- | --- | --- | --- |
|  | 1 | 2 | 3 | 4 | 5 | 6 | 7 | 8 |
| Larva 1 | 0.572 | 0.567 | 0.573 | 0.566 | 0.545 | 0.585 | 0.521 | 0.571 |
| Larva 2 | 0.598 | 0.585 | 0.605 | 0.558 | 0.602 | 0.572 | 0.565 | 0.543 |
| Larva 3 | 0.585 | 0.570 | 0.557 | 0.594 | 0.530 | 0.530 | 0.595 | 0.590 |
| Larva 4 | 0.514 | 0.550 | 0.475 | 0.525 | 0.499 | 0.512 | 0.558 | 0.498 |
| Larva 5 | 0.529 | 0.564 | 0.526 | 0.546 | 0.498 | 0.548 | 0.522 | 0.572 |
| Larva 6 | 0.466 | 0.479 | 0.461 | 0.528 | 0.568 | 0.454 | 0.464 | 0.488 |
| Larva 7 | 0.485 | 0.460 | 0.503 | 0.502 | 0.435 | 0.476 | 0.454 | 0.485 |
| Larva 8 | 0.528 | 0.526 | 0.514 | 0.528 | 0.556 | 0.436 | 0.498 | 0.485 |
| Larva 9 | 0.492 | 0.519 | 0.514 | 0.484 | 0.487 | 0.560 | 0.496 | 0.488 |
| Larva 10 | 0.465 | 0.497 | 0.538 | 0.459 | 0.525 | 0.521 | 0.538 | 0.494 |
