# Supplementary material for: Optimisation of Embryonic and Larval ECG Measurement in Zebrafish for Quantifying the Effect of QT Prolonging Drugs
Source: PLoS One. 2013 Apr 8;8(4):e60552. doi: 10.1371/journal.pone.0060552 (PMC3620317; doi:10.1371/journal.pone.0060552)
Supplement: Table S8 — Measured QTc intervals following movement of electrode. (DOCX) [file pone.0060552.s015.docx]

|  | Mean QTc interval duration (s) | | |
| --- | --- | --- | --- |
| Sample | Before | +5 µm | +10 µm |
| Larva 1 | 0.5184 | 0.5131 | 0.5159 |
| Larva 2 | 0.5223 | 0.5212 | 0.5153 |
| Larva 3 | 0.5206 | 0.4999 | 0.4905 |
