# Supplementary material for: Optimisation of Embryonic and Larval ECG Measurement in Zebrafish for Quantifying the Effect of QT Prolonging Drugs
Source: PLoS One. 2013 Apr 8;8(4):e60552. doi: 10.1371/journal.pone.0060552 (PMC3620317; doi:10.1371/journal.pone.0060552)
Supplement: Table S9 — Effect of terfenadine on QTc interval duration. (DOCX) [file pone.0060552.s016.docx]

| Concentration of terfenadine (µM) | Mean QTc interval duration (s) | |
| --- | --- | --- |
|  | Before | After |
| 0.1 | 0.482 | 0.536 |
| 0.3 | 0.467 | 0.520 |
| 10 | 0.488 | 0.603 |
| 30 | 0.488 | 0.646 |
| 50 | 0.503 | 0.689 |
| *n = 8 per concentration* | | |
