# Supplementary material for: Optimisation of Embryonic and Larval ECG Measurement in Zebrafish for Quantifying the Effect of QT Prolonging Drugs
Source: PLoS One. 2013 Apr 8;8(4):e60552. doi: 10.1371/journal.pone.0060552 (PMC3620317; doi:10.1371/journal.pone.0060552)
Supplement: Table S10 — Effect of haloperidol on QTc interval duration. (DOCX) [file pone.0060552.s017.docx]

| Concentration of haloperidol (µM) | Mean QTc interval duration (s) | |
| --- | --- | --- |
|  | Before | After |
| 50 | 0.499 | 0.586 |
| 100 | 0.511 | 0.603 |
| 200 | 0.504 | 0.593 |
| 300 | 0.480 | 0.576 |
| *n = 8 per concentration* | | |
