# Supplementary material for: Optimisation of Embryonic and Larval ECG Measurement in Zebrafish for Quantifying the Effect of QT Prolonging Drugs
Source: PLoS One. 2013 Apr 8;8(4):e60552. doi: 10.1371/journal.pone.0060552 (PMC3620317; doi:10.1371/journal.pone.0060552)
Supplement: Table S12 — Effect of verapamil on QTc interval duration. (DOCX) [file pone.0060552.s019.docx]

| Concentration of verapamil (µM) | Mean QTc interval duration (s) | |
| --- | --- | --- |
|  | Before | After |
| 10 | 0.482 | 0.486 |
| 25 | 0.478 | 0.484 |
| 50 | 0.471 | 0.529 |
| 100 | 0.473 | 0.578 |
| 200 | 0.484 | 0.591 |
| 300 | 0.477 | 0.622 |
| 400 | 0.481 | 0.609 |
| 1000 | 0.466 | 0.616 |
| *n = 8 per concentration* | | |
