# Supplementary material for: Optimisation of Embryonic and Larval ECG Measurement in Zebrafish for Quantifying the Effect of QT Prolonging Drugs
Source: PLoS One. 2013 Apr 8;8(4):e60552. doi: 10.1371/journal.pone.0060552 (PMC3620317; doi:10.1371/journal.pone.0060552)
Supplement: Table S13 — Effect of penicillin on QTc interval duration. (DOCX) [file pone.0060552.s020.docx]

| Concentration of penicillin (µM) | Mean QTc interval duration (s) | |
| --- | --- | --- |
|  | Before | After |
| 0.1 | 0.497 | 0.495 |
| 0.3 | 0.487 | 0.492 |
| 10 | 0.490 | 0.500 |
| 30 | 0.494 | 0.503 |
| 50 | 0.489 | 0.488 |
| 100 | 0.506 | 0.508 |
| 200 | 0.478 | 0.483 |
| 300 | 0.457 | 0.454 |
| 400 | 0.514 | 0.507 |
| 1000 | 0.511 | 0.501 |
| *n = 8 per concentration* | | |
